# Splash Basins in the Operating Room: Clean or Contaminated? A Study on Bacterial Contamination in Splash Basins Used to Rinse Surgical Instruments During Surgery

**DOI:** 10.3390/nursrep14040296

**Published:** 2024-12-17

**Authors:** Karoline Stavang Michalsen, Linda Helen Helgeland, Grethe Myklestul Dåvøy, Marit Hegg Reime, Fred-Ivan Kvam

**Affiliations:** 1 Oslo University Hospital, Ullevål, Kirkeveien 166, 0450 Oslo, Norway; kamich@ous-hf.no; 2 Haukeland University Hospital, Haukelandsveien 22, 5021 Bergen, Norway; linda.helen.helgeland@helse-bergen.no; 3 Department of Health and Caring Sciences, Faculty of Health and Social Sciences, Western Norway University of Applied Sciences, Inndalsveien 28, 5063 Bergen, Norway; grethe.davoy@hvl.no (G.M.D.); marit.hegg.reime@hvl.no (M.H.R.); 4 Department of Postgraduate Studies, Lovisenberg Diaconal University College, Lovisenberggata 15B, 0456 Oslo, Norway

**Keywords:** surgical site infection, contamination, nursing, operating room, splash basin, surgical instruments, ventilation systems

## Abstract

**Background**: Preventing postoperative infection and promoting patient safety are essential responsibilities of the operating room nurse. In some hospitals, splash basins are used to rinse instruments during surgery, although previous studies emphasise the risk of bacterial contamination. A recent systematic review calls for further investigation into surgical teams’ use of splash basins. **Objectives**: Our objective was to investigate bacterial contamination in splash basins and to identify the variables that may have an influence on this contamination. **Methods**: This prospective observational pilot study involved collecting, cultivating, and analysing water samples obtained from splash basins during operations performed in the thoracic and neurosurgical departments. The ventilation systems, length of surgery, number of instruments in the splash basin, number of persons present in the operating room, frequency of door openings during surgery, and type of bacteria were observed. **Results**: Bacterial growth was found in 44% of the final water samples: 41% from the thoracic surgical department, which had laminar airflow ventilation systems/unidirectional airflow ventilation, and 47% from the neurosurgical department, which had conventional ventilation systems/turbulent mixing ventilation. However, the binary logistic regression analysis revealed no significant correlation between bacterial growth and the ventilation systems, length of surgery, number of instruments in the splash basin, number of people in the operating room, or frequency of door openings. The most common types of bacteria found were coagulase-negative staphylococci and *Micrococcus luteus*. **Conclusions**: Splash basins become contaminated with bacteria during surgery. Therefore, using splash basins with sterile water is not recommended. Further research is needed to determine the best evidence-based practice for rinsing instruments perioperatively.

## 1. Introduction

Surgical interventions increase the patient’s risk of becoming infected, either endogenously by the patient’s bacterial flora or exogenously through the operating room environment [1,2]. Postoperative infections can be a considerable burden for the affected patient. For hospitals and society, this also entails increased expenses [2]. An essential responsibility of the operating room (OR) nurse is the prevention of postoperative infections and the promotion of patient safety [3]. In part, this involves identifying potential sources of contamination and eliminating them [1,2]. Therefore, it is crucial to be knowledgeable about the chain of infection. If the chain is broken in one or more of its links, infection can be avoided [1,2].

The OR nurse is responsible for the operating room environment and the instruments during surgery. In some cases, this involves rinsing the instruments with a splash basin filled with sterile water. The splash basin is used for dipping the instruments and sometimes leaving them to soak. It is also used for dipping gauze to wipe the instruments. This perioperative cleaning procedure can reduce the risk of corrosion and prolong the instrument’s life [3]. The splash basin is a reusable sterile bowl of steel that is decontaminated and sterilised by an autoclave after each use.

However, the research regarding how splash basins can be a source of contamination and transmit bacteria to the patient is somewhat ambiguous. A study by Anto et al. [4] found a 23.8% contamination rate in 21 splash basins and concluded that they should not be used during surgery. In contrast, Christensen et al. [5] and Glait et al. [6] concluded that the risk is lower than assumed, with Glait reporting the lowest contamination rate (2.2%). Lindgren et al. [7] and Nazal et al. [8] conducted randomised controlled trials showing that chlorhexidine or povidone-iodine solutions in the splash basin instead of sterile water significantly decreased bacterial growth. Baird et al. [9] included patients with and without infection before surgery and found a 74% contamination rate in randomly selected cases in both groups. The study by Andersson et al. [10] stands out in its use of saline (irrigation water) instead of sterile water, finding contamination in 13 out of 21 tests (61.9%). Jonsson et al. [11] used swabbing to retrieve bacterial samples to examine bacterial contamination in surgical wounds in orthopaedic prosthetic surgery. Samples were retrieved from the acetabulum before and after cementing, from the fascia after closing, and from the splash basin. Their results showed the highest contamination rate (24.1%) in the samples retrieved from the splash basin. A systematic review of the uses of the splash basin and its infectious potential in orthopaedic surgery calls for further investigation into the surgical teams’ use of and the content of splash basins to advance the understanding of how to make optimal use of this surgical tool [12].

Previous research on bacterial growth in specimens taken from splash basins has studied the associations with different variables, such as the length of surgery [4,6,8,9,10], number of people present in the operating room [4,9,10], and different types of ventilation systems, such as conventional overpressure ventilation (COV)/turbulent mixing ventilation (TMV) and laminar airflow (LAF)/unidirectional airflow (UDAF) ventilation [4,5,9]. In the hospital where this study was conducted, new operating rooms are built with LAF/UDAF ventilation systems, under the assumption that LAF/UDAF ventilation contributes to a lower airborne bacterial load in the OR compared to COV/TMV. The purpose of both ventilation systems is to reduce contamination of the surgical field and sterile equipment like the splash basin [13]. However, a report from the Norwegian Institute of Public Health found no difference in hospital infections when comparing LAF/UDAF ventilation and COV/TMV [14]. Therefore, this variable is emphasised also in the present study to investigate its potential influence on contamination in splash basins.

The research has found coagulase-negative staphylococci to be the most frequently occurring bacteria in splash basins [4,5,8,9]. In earlier studies on splash basins, there was an ultra-clean environment during orthopaedic surgery. Despite the different setups for these studies, they all concluded by recommending that splash basins should not be used in the operating field due to its potential source of contamination [4]. This recommendation is also supported by the consensus of a working group discussing various operating room issues [15].

In Norway, the experience is that some OR nurses reuse instruments several times after soaking them in sterile water. The work performed by OR nurses should be based on evidence-based practice, wherein the research should be incorporated into clinical practice [16]. It is therefore essential to investigate whether splash basins are contaminated with bacteria also in a Norwegian OR context. We want to examine whether the different bacteria found in splash basins are considered pathogenic for humans. In addition to the variables of ventilation systems, length of surgery, and number of persons present in the operating room, this study also includes two more variables not emphasised in previous studies: number of instruments in the splash basin and the influence of the frequency of door openings during surgery. The results of our research may strengthen the knowledge base available when guidelines and procedures are to be implemented regarding the use of splash basins.

### Objective

Although previous studies indicate that splash basins are a potential source of contamination and recommend not using them, they remain in use in some hospitals. The research questions answered in this study are as follows:

- When splash basins are used for rinsing instruments during surgery, do they become contaminated with bacteria? If so, what kinds of bacteria are most present, and are they pathogenic?
- Do ventilation systems, frequency of door openings, number of instruments in the splash basin, number of people in the operating room, and length of surgery have an influence on the contamination of splash basins?

## 2. Materials and Methods

### 2.1. Design

In this prospective observational pilot study conducted at a Norwegian university hospital in February and March 2019, 64 aliquots were collected from water in splash basins. Water samples obtained from splash basins during surgery were filtered, and the filters were incubated to identify whether any bacteria were present. The splash basins were used in the same way during each surgery, namely, for dipping the instruments and sometimes leaving them to soak, and for dipping gauze to wipe the instruments. We used a data collection form to register variables such as operating room facilities with different ventilation systems, length of surgery, number of instruments in the splash basin, number of people in the operating room, and frequency of door openings. These specific variables were chosen based on information in previous studies [4,5,6,8,9,10] and the assumption of potential influence on contamination in splash basins. The Revised Standards for Quality Improvement Reporting Excellence (SQUIRE 2.0) checklist was used for a critical assessment of this study [17].

### 2.2. Setting

This study was conducted in two surgical departments with different facilities and ventilation systems: one had LAF/UDAF ventilation, and the other had COV/TMV. The department with LAF/UDAF ventilation performed thoracic surgery, and the department with COV/TMV performed neurosurgery. The ORs are approximately the same size, and the staff in both departments had the same basic training and used the same type of sterile surgical clothing, including hoods.

### 2.3. Sample Size and Inclusion and Exclusion Criteria

In total, 64 aliquots were collected from water in splash basins from 32 operations. Seventeen of these operations were performed in thoracic LAF/UDAF-ventilated operating rooms and 15 in neurosurgical operating rooms with COV/TMV. Two aliquots were collected from each surgery: one at the start and one at the end. The inclusion criteria were that the splash basins had to be from elective operations lasting 45 to 300 min. Patients with known infections and those requiring emergency surgery were excluded from the study due to the anticipated higher bacterial load and the lack of sufficient time to adhere to established procedures.

### 2.4. Sampling

The quantity of sterile water routinely used in splash basins during surgery was 1000 mL. Two 100 mL aliquot samples were taken from the splash basin: one before the surgical incision and the other immediately after the closure of the wound [4,9]. The samples taken before the surgical incision were retrieved immediately after the splash basins were filled and before any instruments encountered the sterile water. The samples were obtained sterilely in syringes (2 × 50) mL and secured with a plug. The syringes were stored in a cooling box and transported directly to the laboratory. All aliquots were filtered through sterile plastic cups with an MCE filter (mixed cellulose ester) according to the ISO standard [18]. The filter had a diameter of 47 mm and pore size of 0.45 μm [18]. The filter was placed on blood agar and incubated aerobically for approximately 48 h at 36–37 °C, and the visual colonies were counted. Matrix-assisted laser desorption time-of-flight mass spectrometry (MALDI-TOF MS) was performed at the microbiological unit at the same hospital where the samples were collected to identify the different bacteria [19]. If the colonies looked similar, only some were chosen for identification for capacity reasons.

### 2.5. Data Collection and Variables

A data collection form was designed to register the various variables. Variables such as the ventilation systems, length of surgery, number of instruments in the splash basin, number of people present in the operating room (both in a sterile gown and circulating), and frequency of door openings were registered. The data registration started when the sterile water was poured into the splash basin, parallel to the first aliquot sample being taken before the surgical incision. The circulating personnel were registered only once, even if they left the room and came back in. Nevertheless, this was captured as all instances of door openings during the surgery were registered. The data registration stopped when the last aliquot was taken immediately after closing the wound.

### 2.6. Statistical Analysis

We used frequency counts, percentages, means, standard deviations (SD), and interquartile ranges (IQR) for descriptive statistics. To compare the two ventilation systems, we used the chi-square test, an independent *t*-test, and the Mann–Whitney rank sum test. Binary logistic regression analysis determined the influence of the different registered variables on bacterial contamination. The analysis was performed using Stata 14 and SPSS version 22/23; *p*-values ≤ 0.05 were considered statistically significant.

## 3. Results

In samples taken before the surgical incision, bacterial growth was shown in five samples (16%; not presented in a table). Results from the aliquots obtained at the end of surgery, comparing the two differently ventilated facilities, are shown in Table 1.

The results reveal a total contamination rate of 44% in the splash basins. A comparison of the two ventilation systems shows bacterial growth in 41% of the samples from the LAF/UDAF-ventilated rooms and in 47% of those from the COV/TMV rooms. However, the *p*-value indicates no statistical significance between the two ventilation systems regarding bacterial growth. Only the variables “number of people present in the operating room in a sterile gown”, “frequency of door openings”, and “length of surgery” were statistically significant between the two different ventilated facilities. Comparison of “number of people circulating” and “number of instruments in the splash basin” showed no statistical difference between the facilities.

When using binary logistic regression analysis, none of the investigated variables or combinations significantly affected bacterial contamination in the splash basins (Table 2).

In each sample, few bacterial colonies were detected. The most common result was one colony per 100 mL sample. Some samples showed up to three colonies, one sample showed seven, and two samples taken after the wound was closed showed 55 and 55+/uncountable (as the colonies were too close to each other to count correctly). When investigating colonies in relation to the different ventilation systems, the median colony count was 1 in the LAF/UDAF-ventilated rooms and 2 in ORs with COV/TMV. *Staphylococcus epidermidis* was the bacteria found in most samples and was present in seven of the samples taken after wound closing. *Micrococcus luteus* was found in five samples, and *Staphylococcus capitis* was found in four samples (Table 3).

## 4. Discussion

This study aimed to determine whether splash basins used to rinse and wipe instruments during surgery become contaminated with bacteria, which kinds of bacteria, and if they are pathogenic. It also aimed to identify the variables that can affect this outcome, such as the ventilation systems, frequency of door openings, number of instruments in the splash basin, number of people in the operating room, and length of surgery.

### 4.1. Do Splash Basins Become Contaminated During Surgery?

Bacterial growth was detected in 44% of the final samples, which is in accordance with several previous studies [4,7,9]. Although we found bacteria in a large proportion of the final samples, few colonies were counted in most of these, giving the impression that even if the water in the splash basin was contaminated, it was not heavily contaminated. However, the results may be inaccurate, because no known precise cultivation method exists for growing all types of bacteria under laboratory conditions [20]. Under these conditions, one would not expect more than 0.01–10% of the bacteria to grow [20]. Hypothetically, it is possible that the splash basins contained more bacteria than we found, as we did not filter all of the water in the splash basins, which leaves a scope for some bacteria in the water to be missed. In the study by Andersson et al. [10], the entire residual amount from the splash basin was filtered, and a contamination rate of 61.9% was found.

### 4.2. Do the Different Ventilation Systems Have Influence on Bacterial Contamination of Splash Basins?

The two ventilation systems did not significantly differ in influencing bacterial contamination in the splash basins. However, the median colony count was 1 in the LAF/UDAF-ventilated rooms and 2 in ORs with COV/TMV. Our results show an almost equal number of positive samples, with 41% from LAF/UDAF-ventilated ORs and 47% from ORs with COV/TMV. This is somewhat surprising since there is a significant difference between the departments when it comes to the variables number of people present in the OR, frequency of door openings, and length of surgery. Due to our small sample size, our results should be interpreted with caution regarding the impact of the different ventilation systems on bacterial contamination of the splash basins. However, a systematic review and meta-analysis also found no benefit of LAF/UDAF ventilation compared with COV/TMV in the OR in reducing the risk of surgical site infections [21]. In contrast, in their comparison study of mixed and laminar airflow systems, Andersson et al. [22] showed that LAF/UDAF ventilation lowers the bacterial burden in OR air and, specifically, that LAF/UDAF ventilation systems offer low bacterial levels proximate to a surgical wound during surgery, which is also supported by findings from other studies [23,24].

### 4.3. Can Bacteria Detected from Splash Basins Lead to Infection?

Patient resilience, virulence of the bacteria, and the number of bacteria present are relevant in developing postoperative wound infections [2]. *S. epidermidis* is considered the most important opportunistic pathogen and is a frequent cause of hospital-related infections [25]. As *S. epidermidis* permanently colonises the skin, the risk of contamination increases when surgical instruments penetrate the skin barrier. Infection with *S. epidermidis* rarely becomes life-threatening, but the frequency of its occurrence and the complexity of treatment due to resistance development make this type of infection very costly [25]. *M. luteus* is also typically found in the skin flora and is an opportunistic bacterium. It can lead to hospital-related infections and is often clinically confused with *S. aureus*. In immunosuppressed patients, *M. luteus* may cause septic shock [26]. *S. capitis* can pose a risk to newborns and patients with weakened immune systems, and cases have been described in which *S. capitis* is the cause of endocarditis [27] and the postoperative infection of orthopaedic prostheses [28]. The bacteria found in the splash basin can lead to varying degrees of infection, but we cannot predict the outcome in advance. We can, however, show that these types of bacteria have been detected in postoperative infections [25,26,27,28,29]. Our bacterial findings resemble the findings described in the other studies we have presented, with the highest incidence being coagulase-negative staphylococci [4,5,8,9].

### 4.4. Is the Degree of Bacterial Contamination of Splash Basins Influenced by the Frequency of Door Openings, the Number of Instruments in the Splash Basin, the Number of People in the Operating Room, and the Length of Surgery?

There was no correlation between bacterial growth and the variables “frequency of door openings”, “number of instruments in the splash basin”, ”number of people present in the operating room”, and” length of surgery” in the binary logistic regression analysis. None of the previous studies on splash basins included the frequency of door openings, but Andersson et al. [22] did include door-opening rates in their comparison study on mixed and laminar airflow systems. They found that the rate of door opening increased the bacterial load in the OR air, regardless of which ventilation system was used, but with a higher burden in COV/TMV rooms than in LAF/UDAF-ventilated rooms. Andersson et al. [10] included the variable number of OR personnel but found no correlation with contamination of the splash basin, which is consistent with our findings. Several previous studies have included the length of surgery [6,7,8,11]. We found no correlation between the length of surgery and the contamination of splash basins in our study, which contrasts with the findings of the studies by Anderson et al. [10], Lindgren et al. [7], Jonsson et al. [11], Nazal et al. [8], and Glait et al. [6], who found a greater risk of contamination with longer operating times. Our results show that the number of instruments in the splash basin varied at the end of surgery. However, the number of instruments did not have an influence on bacterial contamination in the splash basin. Glait et al. [6] also included the number of instruments in the splash basin as a variable but did not report the results in their article.

### 4.5. Strengths and Limitations

A strength of this study is that we ran several tests before the start of the study to ensure we had a valid method for detecting bacteria in filtered water. The method selected is also described in studies by Baird et al. [9] and Anto et al. [4]. Colonies that grew on agar outside the filter were not counted and included in the result, as they were deemed to be contaminated.

This study’s limitations concern the laboratory environment in which the water filtration was carried out, as it cannot be considered sterile, and contamination of the samples may have occurred during sample preparation. The samples taken before the incision showed bacterial growth in 16% of the samples. As splash basins are used as a catcher/drop point for sterile equipment during preparation for surgery, they may have been contaminated before the water was poured into them [30]. For this reason, the aliquot taken before the incision could not serve as a reference point for the samples taken after the wound closure. Difficulties describing the findings of bacterial growth in the initial samples are also referred to in the articles by Glait et al. [6] and Nazal et al. [8]. Glait et al. [6] only mention the findings in the article, and Nazal et al. [8] removed the samples from further analysis. We decided to describe our findings despite our inability to explain the bacterial growth from samples collected before the start of the surgical procedure.

In our study, five samples (16%) taken before the surgical incision showed bacterial growth, all of which were from LAF/UDAF-ventilated operating rooms. For two of the five positive samples, there was no bacterial growth in the associated sample taken after wound closure. These results are not presented in a table but represent an issue for further discussion.

We did not register how frequently the splash basins were used during each surgery to rinse instruments or dip gauze, which is another limitation, but the splash basins were used to some degree in all of the observed surgeries. In addition, the source of contamination of splash basins can be a mix of many factors, both from the environment or the patient himself [2], which makes it difficult to conclude how bacteria ended up in the splash basin.

Our study had time and resource limitations, which narrowed the sample size. The small sample size may entail a risk of type 2 errors when performing regression analysis. We might have had different results with a larger sample size, and we recommend that future studies conduct a power analysis in advance.

### 4.6. Suggestions to Prevent Splash Basins from Transmitting Bacteria to Sterile Instruments

To prevent sterile instruments from being contaminated by bacteria in splash basins when they are rinsed or reused, it may be necessary to open sterile instruments from new packaging. The research should be conducted on alternative methods of rinsing surgical instruments perioperatively, such as using a wet gauze to wipe the instruments and discard them after wiping or using closed systems for the sterile water to clean instruments. Using splash basins containing various antibacterial agents is another intriguing method that warrants further investigation in experimental studies of good quality.

In a broader perspective, a number of procedures are used to reduce the number of microbes in the operating theatre environment and thereby prevent surgical infections. Ultraviolet-C (UVC) technology has been known for a long time and is now being tested in operating theatres to reduce the number of pathogenic microbes [31].

## 5. Conclusions

The total contamination rate of the splash basins was 44%. This high rate makes the splash basin a source of contamination during surgery. Moreover, as the results show, the types of bacteria we found may lead to infection. There was no significant correlation between bacterial growth and the variables length of surgery, number of instruments in the splash basin, number of people in the operating room, frequency of door openings, and the different ventilation systems.

Our results correspond with previous studies, suggesting that instruments placed in splash basins should not be reused during surgery.

## Figures and Tables

**Table 1:** Results from the aliquots obtained at the end of surgery. A comparison of two surgical departments with different ventilation facilities at a Norwegian university hospital.

Variables	All (n = 32)	LAF/UDAF Ventilation (n = 17)	COV/TMV (n = 15)	*p*-Value
Bacterial growth, n (%)	14 (44)	7 (41)	7 (47)	0.760 ^a^
Length of surgery (minutes), mean (SD)	156 (74)	200 (60)	107 (57)	<0.001 ^b^
People present in a sterile gown, mean (IQR)	3 (3, 4)	4 (4, 5)	3 (3, 3)	<0.001 ^c^
People circulating, mean (IQR)	7 (6, 9)	7 (7, 9)	6 (6, 9)	0.580 ^c^
Door openings (per operation), mean (IQR)	46 (34, 62)	48 (44, 64)	32 (27, 54)	0.023 ^c^
Instruments in the splash basin, mean (IQR)	11 (15, 16)	15 (4, 16)	10 (5, 18)	0.980 ^c^

Abbreviations: LAF = laminar airflow; UDAF = unidirectional airflow; COV = conventional overpressure ventilation; TMV = turbulent mixing ventilation; SD = standard deviation; IQR = interquartile range; ^a^ chi-square test; ^b^ independent *t*-test; ^c^ Mann–Whitney rank sum test.

**Table 2:** Results from binary logistic regression analysis of bacterial growth in splash basins with respect to six potential sources of contamination.

	Unadjusted Models				Fully Adjusted Model (n = 31)		
Sources	n	OR	95% CI	*p*	OR	95% CI	*p*
Sterile persons present, n	32	1.04	(0.54, 1.99)	0.901	0.84	(0.26, 2.68)	0.763
Unsterile persons present, n	32	1.06	(0.79, 1.42)	0.677	1.05	(0.67, 1.67)	0.825
Door openings, per 10 min	32	1.12	(0.75, 1.67)	0.590	0.78	(0.36, 1.71)	0.530
Instruments in splash basin, per 10 min	31 *	1.19	(0.41, 3.51)	0.722	1.06	(0.32, 3.56)	0.922
Length of surgery, per 10 min	32	1.03	(0.93, 1.13)	0.559	1.12	(0.91, 1.37)	0.268
COV/TMV vs. LAF/UDAF ventilation	32	1.25	(0.31, 5.07)	0.755	1.71	(0.18, 18.09)	0.652

Abbreviations: OR = odds ratio; CI = confidence interval, *p* = from likelihood ratio test; LAF = laminar airflow ventilation; UDAF = unidirectional airflow ventilation; COV = conventional overpressure ventilation; TMV = turbulent mixing ventilation; * data missing from one operation.

**Table 3:** Number of samples with the different types of bacteria identified before and after surgery.

Bacteria	Samples Before Surgery	Samples After Surgery
*Staphylococcus epidermidis*		7
*Staphylococcus hominis*	1	1
*Micrococcus luteus*	2	5
*Staphylococcus capitis*		4
*Staphylococcus haemolyticus*		1
*Bacillus thuringiensis/cereus/Bacillus species*		1
*Corynebacterium lipophiloflavum/Corynebacterium species*	1	1
No peaks found	1	2

## Data Availability

The data that support the findings of this study are available from the corresponding author upon reasonable request.

## Public Involvement Statement

No public involvement in any aspect of this research.

## Guidelines and Standards Statement

The Revised Standards for Quality Improvement Reporting Excellence (SQUIRE 2.0) checklist was used for a critical assessment of this study [17].
